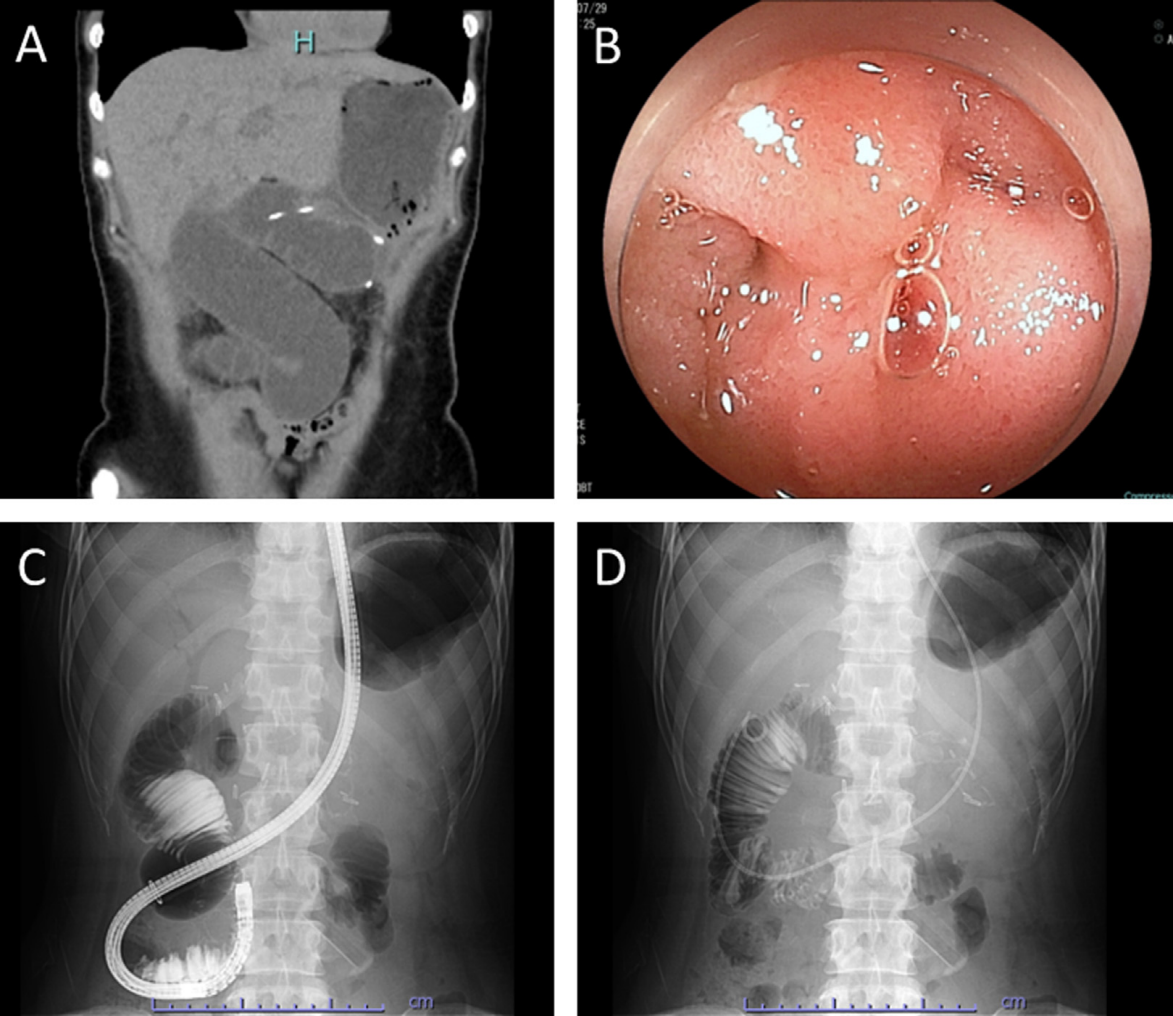# A Rare Cause of Afferent Loop Syndrome

**DOI:** 10.1016/j.gastha.2022.10.009

**Published:** 2022-10-22

**Authors:** Kosei Takagi, Nanako Hata, Yuki Fujii

**Affiliations:** 1 Department of Gastroenterological Surgery, Okayama University Graduate School of Medicine, Dentistry, and Pharmaceutical Sciences, Okayama, Japan; 2 Department of Gastroenterology, Okayama University Hospital, Okayama, Japan

A 47-year-old female underwent robotic pancreatoduodenectomy for the pancreatic head cancer. During surgery, the jejunum (the afferent limb) was brought up, although the Treitz ligament route to perform the pancreaticojejunostomy and hepaticojejunostomy anastomoses, followed by the antecolic gastrojejunostomy. The patient presented with acute abdominal pain 3 months after surgery. Laboratory found the elevated hepato-pancreato-biliary enzymes: Total bilirubin, 2.0 mg/dL; aspartate aminotransferase, 650 U/L; alanine aminotransferase, 406 U/L; and amylase, 178 U/L. Computed tomography showed the swollen afferent loop (Figure A). Double-balloon enteroscopy was performed for the treatment and revealed the closed loop due to the afferent limb volvulus (Figure B and Figure C), which was successfully repositioned with the endoscopic intervention (Figure D). The patient was followed up without recurrence of the afferent loop syndrome for a few months.

Afferent loop syndrome due to the afferent limb volvulus is a rare complication following gastrectomy with a Billroth II or Roux-en-Y reconstruction. To the best our knowledge, this is the first report to demonstrate afferent loop syndrome by the afferent limb volvulus after robotic pancreatoduodenectomy. Diagnostic and therapeutic endoscopic intervention can be the first option. Next, surgical intervention should be considered in complicated cases refractory to the endoscopic intervention.

**Figure:**